# Measuring the contribution of built-settlement data to global population mapping

**DOI:** 10.1016/j.ssaho.2020.100102

**Published:** 2021

**Authors:** Jeremiah J. Nieves, Maksym Bondarenko, David Kerr, Nikolas Ves, Greg Yetman, Parmanand Sinha, Donna J. Clarke, Alessandro Sorichetta, Forrest R. Stevens, Andrea E. Gaughan, Andrew J. Tatem

**Affiliations:** a WorldPop, School of Geography and Environmental Science, University of Southampton, UK; b Center for International Earth Science Information Network (CIESIN), Columbia University, Palisades, NY, USA; c Department of Geography and Geosciences, University of Louisville, Kentucky, USA

**Keywords:** Urban, Population, Growth model, Built, Settlement, Machine learning, Meta-analysis

## Abstract

Top-down population modelling has gained applied prominence in public health, planning, and sustainability applications at the global scale. These top-down population modelling methods often rely on remote-sensing (RS) derived representation of the built-environment and settlements as key predictive covariates. While these RS-derived data, which are global in extent, have become more advanced and more available, gaps in spatial and temporal coverage remain. These gaps have prompted the interpolation of the built-environment and settlements, but the utility of such interpolated data in further population modelling applications has garnered little research. Thus, our objective was to determine the utility of modelled built-settlement extents in a top-down population modelling application. Here we take modelled global built-settlement extents between 2000 and 2012, created using a spatio-temporal disaggregation of observed settlement growth. We then demonstrate the applied utility of such annually modelled settlement data within the application of annually modelling population, using random forest informed dasymetric disaggregations, across 172 countries and a 13-year period. We demonstrate that the modelled built-settlement data are consistently the 2nd most important covariate in predicting population density, behind annual lights at night, across the globe and across the study period. Further, we demonstrate that this modelled built-settlement data often provides more information than current annually available RS-derived data and last observed built-settlement extents.

## 1 Introduction

Human settlement and population dynamics are more important than ever to understand (Ehrlich, Balk, & Sliuzas, 2020; Zhu et al., 2019) as an additional 13 percent of the world’s population will live in urbanized areas by 2050, with most of this growth occurring in low-to middle-income countries (Angel, Parent, Civco, Blei, & Potere, 2011; United Nations, 2018). Most of this projected growth will not occur in the largest cities, but rather it will occur in small to medium sized settlements (Cohen, 2004), which are typically underrepresented in various measures and counts including censuses (Leyk et al., 2019; Tatem, Noor, von Hagen, Di Gregorio, & Hay, 2007) and remote-sensing (RS)-derived representations of settlements (Kuffer, Barros, & Sliuzas, 2014; Kuffer, Pfeffer, & Sliuzas, 2016; Nieves et al., 2020; Pesaresi et al., 2013; Weber et al., 2018). This projected growth has implications for sustainable development (Ehrlich et al., 2020), which has been noted in the 2030 Sustainable Development Goals (SDGs) (United Nations, 2016).

The rapid rate of growth and magnitude of the urbanization of populations, and land cover transitions from more natural to more built, requires greater data and information about urban areas and human settlement, including higher frequency of urban areas observations (Hoalst-Pullen & Patterson, 2011a; Zhu et al., 2019). These data demands are, in part, driven by broader motivations similar to the SDG aim of making sure “no one is left behind” (United Nations - Economic, 2016), with a specific goal to expand the availability and accessibility of base data to help facilitate the planning, implementation, and assessment of programs and applications to achieve the 2030 SDGs (Scott & Rajabifard, 2017; United Nations, 2016). These applications reliant upon built-environment and settlement data, equally require time-specific population maps between decadal censuses (Balk et al., 2006a; Bhaduri, Bright, & Coleman, 2007; Leyk et al., 2019; Tatem, 2014; Tatem et al., 2007), for planning purposes and to monitor progress of interventions or policy effects (Bharti, Djibo, Tatem, Grenfell, & Ferrari, 2016; Juran et al., 2018; Linard et al., 2017; McGranahan, Balk, & Anderson, 2007; Patel et al., 2015; Tatem, 2018). To meet the demand for time-specific population maps, top-down population models are often utilized and are frequently dependent upon data on or relating to human settlement to inform their disaggregation of populations across space (Freire, MacManus, Pesaresi, Doxsey-Whitfield, & Mills, 2016; Leyk et al., 2019; Nieves et al., 2017a). As such, the demand for time-specific and consistently defined built-environment data is doubly so (Ehrlich et al., 2020; Gaughan et al., 2016; Henderson, Yeh, Gong, Elvidge, & Baugh, 2003; Zhu et al., 2019).

Remote Sensing (RS) would naturally be an answer to such data needs. However, most change detection algorithms and urban related studies have disproportionately focused on larger cities, particularly those within the US, Europe, and China raising questions of representativeness (Acuto, Parnell, & Seto, 2018; Seto, Fragkias, Guneralp, & Reilly, 2011; Zhu et al., 2019). However, since 2010, a new class of globally available and consistent RS-derived representations of built-settlement, have become available at single and multiple time points (Corbane et al., 2017a; Esch et al., 2013; Esch et al., 2018a; Facebook Connectivity Lab, 2016; Microsoft.BuildingFoot, 2018; Pesaresi et al., 2013; Pesaresi et al., 2016). Built-settlement (BS) is defined as above ground structures that can support human habitation and related economic phenomena (Florczyk et al., 2019; Nieves et al., 2020; Pesaresi et al., 2013). The concept of BS addresses the “distribution of buildings by which people attach themselves to the land” (Ehrlich et al., 2020; Stone, 1965) and these data are better able to differentiate between buildings and other aspects of the built environment, such as road ways or parking lots. This new class of data is also better able to capture small settlements due to having higher spatial resolutions, typically ranging from the representation of individual buildings to 50m (Esch et al., 2013; Pesaresi et al., 2013; Pesaresi et al., 2016; Zhu et al., 2019), and have been found to be highly important in top-down population modelling applications (Leyk et al., 2019; Nieves et al., 2017a; Patel et al., 2015; Reed et al., 2018; Stevens et al., 2020). These characteristics, capturing even small settlements and having a definition more closely tied to populations, make this class of data particularly well suited for top-down disaggregation models of population.

These new data would seem to address the call for urban data, as Zhu et al. (Zhu et al., 2019) summarize, “be consistent and harmonized for boundaries, comparable across cities and over time”, particularly as applied to where humans may locate for either habitation or economic activities. However, rapidly changing landscapes, particularly within and around urban areas, requires a higher frequency of coverage as well as longer temporal record (Hoalst-Pullen and Patterson, 2011b; Zhu et al., 2019), which these data, such as the Global Human Settlement Layer (Corbane et al., 2017b; Pesaresi et al., 2013), the Global Urban Footprint (Esch et al., 2013), and the World Settlement Footprint (Marconcini et al., 2020), currently lack. Additionally, Like most RS-derived products, they are limited by the quality and availability of imagery, training and validation data, atmospheric conditions, and sensor/platform errors (Corbane et al., 2017a; Esch et al., 2013; Esch et al., 2018a; Pesaresi et al., 2013; Pesaresi et al., 2016). There is also a substantial lag between the collection of imagery and the production of these datasets (Zhu et al., 2019). While these new datasets have leveraged advances in imagery availability, computational resources, and statistical methods, the processes to produce these finished BS datasets are still computationally expensive (Cheriyadat, Bright, Potere, & Bhaduri, 2007; Esch et al., 2018a; Esch et al., 2018b).

The aforementioned settlement and population modelling needs, combined with the current temporal limits of BS datasets, have prompted some to interpolate BS extents in a globally consistent manner (Nieves et al., 2020). These efforts produce annually estimated BS extents while expanding the temporal frequency and coverage of these BS datasets while maintaining its dataset specific definition of BS (Nieves et al., 2020). A larger question, accompanying any further application of modelled data, is how these interpolated settlement extents contribute to subsequent modelling applications when lacking comparison to a ground truth. To the best of our knowledge, no large-scale assessment of the potential contributions of urban/settlement/built-environment growth model outputs to subsequent models has been undertaken. This is particularly so for assessing the potential impact of utilizing modelled BS extents in time-specific modelling population distributions. Lacking time specific BS extent data, top-down, i.e. disaggregative, population modelling applications typically utilize the last observed RS-derived built-environment extents (Balk, Pozzi, Yetman, Deichmann, & Nelson, 2004; Balk et al., 2006b).

Here, we examine the utility of interpolated BS extent data within a top-down population modelling context. This examination takes the form of a meta-analysis of covariate importances extracted from the population models with a specific focus on how the modelled BS extents were or were not important in the population modelling process. Specifically, within this work, we seek to address whether modelled BS extents were more informative than the last observed BS extents, when both are provided as covariates to the population model. Additionally, we investigated if time-specific modelled BS extents are important to population models of this type and if this importance varied across region and time. Lastly, we explored the relative contribution of time-specific modelled BS extents to time-specific RS-derived BS extents and see if their importance in population modelling varied by region and across time.

## 2 Materials and methods

To begin to examine how modelled BS could contribute meaningfully to population modelling applications, we examined 4662 annual country-specific disaggregative population model objects of the WorldPop “Global Project” (WorldPop - School of Geog, 2018) from 2000 through 2020. These model objects were constructed from subnational census-based population counts and estimates from 2000-2020 (Doxsey-Whitfield et al., 2015) and were specific to each country and year. Specifically, we looked at a subset of these model object (*n ​=* 2236) where BS extents were annually interpolated (Nieves et al., 2020) globally between 2000-2012 and subsequently used as a covariate within a random forest-informed population disaggregation model (Stevens, Gaughan, Linard, & Tatem, 2015). The built-settlement modelling framework, which we present an overview of here, has been previously described in the literature (Nieves et al., 2020). These BS covariates, within the population models, included an annually modelled BS extents covariate, an annually available RS-derived BS extents covariate, and a single-year “historical” BS extents covariate corresponding to the year 2000. We performed a meta-analysis (Nieves et al., 2017a) of the covariate importance of the annually modelled BS extents covariate, relative to all other covariates, in modelling population density through a top-down disaggregative framework.

### 2.1 Study area

**Fig. 1:**
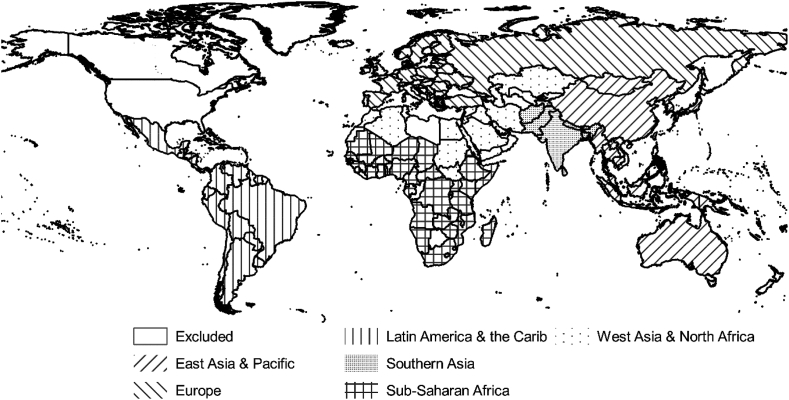
Map of countries included in the meta-analysis and the regional groups used in analyses. See Appendix A, Table 2 for a list of countries excluded from analyses and corresponding exclusion criteria.

Here, we examined population models from 222 countries across the years 2000–2020. We then subset these models and countries to only include the interpolative years of 2000–2012. Countries were excluded from analysis because they either did not have the BSGM model run (due to resource limitations) or they were modelled using a regional model parameterization, similar to Gaughan et al. (Gaughan, Stevens, Linard, Patel, & Tatem, 2014), resulting in 172 countries for analysis across 13 years resulting in a sample of 2236 country specific model objects for analyses. Regional parametrization precludes any analysis of the country specific importance of any covariates due to the merging of random forest model objects (Table 1) (Nieves et al., 2017b). Of specific note was the exclusion of the USA. We excluded it from this analysis because the BS model was not run on its 10 million plus subnational units and large spatial extent due to project resource limitations. For analyses we adopted a regional grouping of countries initially based upon The World Bank’s regional groupings (The World, 2020), but modified in some areas based upon economic, historical, developmental, and urbanization context similarity/dissimilarity (Fig. 1). Because the “North American” region only included two modelled countries (Canada and Greenland), we excluded it from further analyses. A full list of countries that were modelled and their region grouping is in Appendix A, Table 1 and a list of countries excluded from our analysis, and the corresponding reason, are in Appendix A, Table 2.

**Table 1:** Table of geospatial covariates used in the modelling of annual BS using the interpolative Built-Settlement Growth Model (BSGMi) per Nieves et al. (Nieves et al., 2020). Here, representation of BS here is a combination of ESA, GHSL, and GUF as described in Lloyd et al. (Lloyd et al., 2019).

Covariate	Description	Use a, c	Time Point(s)	Original Spatial Resolution(s) at Equator (approx.)	Data Source(s)
DTE Protected Areas Category 1	Distance To the nearest Edge (DTE) of level 1 protected area	Spatial Allocation^c^	2012	Vector	Enviroment Programme (2015)
Subnational Population	Annual population by sub-national units	Demand Quantification	2000–2020, annually	Vector	Doxsey-Whitfield et al. (2015)
Built-settlement^b^	Binary BS extents	Demand Quantification and Spatial Allocation	2000 2012	30m, 60m, & 300m	(Esch et al., 2013; European Space A, 2017; Pesaresi et al., 2013)
DTE Built-settlement	Distance to the nearest BS edge	Spatial Allocation^c^	2000	30m, 60m, & 300m	(Esch et al., 2013; European Space A, 2017; Pesaresi et al., 2013)
Proportion Built-settlement 1,5,10,15	Proportion of pixels that are BS within 1,5,10, or 15 pixel radius	Spatial Allocation^c^	2000	30m, 60m, & 300m	(Esch et al., 2013; European Space A, 2017; Pesaresi et al., 2013)
Elevation	Elevation of terrain	Spatial Allocation^c^	2000 – Time Invariant	90m	Lehner, Verdin, and Jarvis (2008)
Slope	Slope of terrain	Spatial Allocation^c^	2000 – Time Invariant	90m	Lehner et al. (2008)
Water	Areas of water to restrict areas of model prediction	Restrictive Mask		150m	Lamarche et al. (2017)
Weighted Lights-at-Night (LAN)^d^	Annual lagged and sub-national unit-normalised LAN	Spatial Allocation	2000–2011, annually	926m	DMSP (Lloyd et al., 2019; Zhang, Pandey, & Seto, 2016)

a Covariates involved in Demand Quantification were used to determine the demand for non-BS to BS transitions at the subnational unit level for every given year. Covariates involved in Spatial Allocation were either used as predictive covariates in the random forest calculated probabilities of transition (see c) or as a post-random forest year specific weight on those probabilities and the spatial allocation of transitions within each given unit area. Covariates used as restrictive masks prevented transitions from being allocated to these areas.

b The binary BS data utilized 2000 and 2012 as observed points in the dasymetric modelling process, but only derived covariates for 2000 were utilized in the random forest as predictive covariates.

c Used as predictive covariates in the random forest calculated probabilities of transition.

d Readers are referred to Nieves et al. [5] for details on the lagging, normalizing and weighting procedure.

### 2.2 Population data

Annual estimates of subnational population across the globe were provided by the Center for International Earth Science Information Network (CIESIN) and are based upon the work of Gridded Population of the World, version 4 (GPW, v4). Population counts are based upon censuses and/or official estimates which were interpolated to estimate annual counts, following Doxsey-Whitfield et al. (Doxsey-Whitfield et al., 2015). The subnational unit areas (hereafter simply “unit”) were spatially harmonized and assigned a unique identifier corresponding to a globally consistent grid of harmonized coastlines and international borders, as described in Lloyd et al. (Lloyd et al., 2019).

### 2.3 Built-settlement (BS) data

Built-settlement (BS) (Nieves et al., 2020) is based upon the definition put forth by Pesaresi et al. (Pesaresi et al., 2013), “… enclosed constructions above ground which are intended for the shelter of humans, animals, things or for the production of economic goods and that refer to any structure constructed or erected on its site.” This was further generalized by Nieves et al. (Nieves et al., 2020) to include any datasets attempting to better capture buildings and structures within the above definition while attempting to exclude general impervious surface land cover which lacks a vertical dimension (e.g. roads, runways, parking lots), whether this is achieved through a feature extraction process or from post-processing.

Here the input BS data is a combination of the Global Human Settlement Layer (GHSL) 38m settlement extents for the year 2000 (Corbane et al., 2017a; Pesaresi et al., 2013), the “Urban areas” thematic class, class 190, from the ESA CCI land cover 300m global time series for the year 2000 (hereafter ESA) (European Space A, 2017), and the Global Urban Footprint (GUF) 72m settlement extents representing circa 2012 (Esch et al., 2013). These data were resampled to 100m and spatially harmonized as detailed in Lloyd et al. (Lloyd et al., 2019), with the ESA data used, in conjunction with the information supplied by the GUF 2012 information, to systematically back-fill missing portions within large settled areas due to imagery availability and atmospheric conditions. Further, to represent the 2012 time point and facilitate agreement, the backfilled GHSL-ESA 2000 layer was mosaiced, i.e. union, with the GUF 2012. The resulting BS extents, for 2000 and 2012, were used as is to derive covariates for use in predicting the annually interpolated BS extents, 2001 through 2011, and for predicting gridded population surfaces, for their corresponding year of representation.

### 2.4 Geospatial covariates

A suite of geospatial covariates is used in interpolating the annual BS extents as well as disaggregating the annual unit-area population counts into annual gridded population surfaces. All covariates were produced as described in Lloyd et al. (Lloyd et al., 2019), with categorical covariates converted to a continuous covariate, by calculating the Distance-To-nearest-Edge (DTE), for areal type covariates and distance-to-nearest feature calculated for linear and point type covariates. A list of covariates, their original resolution, their source, and a description of them are given in Table 1, Table 2.

**Table 2:** Table of geospatial covariates used in the disaggregative modelling of gridded population surfaces.

Covariate	Variable Name(s) in Random Forest	Description	Time Point(s)	Original Spatial Resolution(s) at the Equator (approx.)	Data Source(s)
DTE Protected Areas Category 1	wdpa_cat1_dst	Distance To the nearest Edge (DTE) of level 1 protected area	2000–2012	Vector	(Enviroment Programme, 2015; Lloyd et al., 2019)
Subnational Population	–	Annual population by sub-national units	2000–2020, annually	Vector	Doxsey-Whitfield et al. (2015)
Distance to OpenStreet Map (OSM) Rivers	osmriv_dst	Distance to nearest OSM river feature	2017	Vector	(Lloyd et al., 2019; OpenStreetMap Contributer, 2017)
Distance to OpenStreet Map (OSM) Road Intersections	osmint_dst	Distance to nearest OSM road intersection feature	2017	Vector	(Lloyd et al., 2019; OpenStreetMap Contributer, 2017)
Distance to OpenStreet Map (OSM) Roads	osmroa_dst	Distance to nearest OSM road feature	2017	Vector	(Lloyd et al., 2019; OpenStreetMap Contributer, 2017)
DTE Built-settlement a, b	ghsl_esa_dst; bsgm_wpgp_dst ghsl_guf_dst; ghsl_esa_dst_2000	Distance To the nearest Edge (DTE) of BS	2000; 2001–2011; 2012; 2001–2012	30m, 60m, & 300m	(Esch et al., 2013; European Space A, 2017; Lloyd et al., 2019; Pesaresi et al., 2013)
Elevation	Topo	Elevation of terrain	2000 – Time Invariant	90m	(Lehner et al., 2008; Lloyd et al., 2019)
Slope	Slope	Slope of terrain	2000 – Time Invariant	90m	(Lehner et al., 2008; Lloyd et al., 2019)
Water	cciwat_dst	Areas of water to mask areas of model prediction and, for inland bodies of water, as a DTE covariate		150m	(Lamarche et al., 2017; Lloyd et al., 2019)
ESA CCI Land Cover (LC) Class^c^	ccilc_dst<*class number*>_<*year*>	Distance To nearest Edge (DTE) of individual land cover classes	2000	300m	(European Space A, 2017; Lloyd et al., 2019)
Lights At Night (LAN)	dmsp; viirs	Annual average of LAN atmospheric radiance	2000–2011; 2012	900m	(Earth Observation Group N, 2013; Lloyd et al., 2019)
Average Precipitation	wclin_prec	Mean Precipitation	1950–2000	900m	(Hijmans, Cameron, Parra, Jones, & Jarvis, 2005; Lloyd et al., 2019)
Average Temperature	wclim_temp	Mean temperature	1950–2000	900m	(Hijmans et al., 2005; Pezzulo et al., 2017)

a ghsl_esa_dst was only used in the year 2000 population model; bsgm_wpgp_dst was derived from the BSGM predicted extents and used for years 2001–2011; ghsl_guf_dst was used for the year 2012.

b ghsl_esa_dst_2000 is identical to ghsl_esa_dst, but was included as a covariate in all models from 2001 onward to avoid unrealistic population distributions as seen in multitemporal modelling within Gaughan et al. (Gaughan et al., 2016).

c Some classes were collapsed: 10–30 ​→ ​11; 40–120 ​→ ​40; 150–153 ​→ ​150; 160–180 ​→ ​160 (Sorichetta et al., 2015).

### 2.5 Methods

#### 2.5.1 Built-settlement growth model interpolation (BSGMi)

The Built-Settlement Growth Model interpolation (BSGMi) is a top-down modelling framework that disaggregates observed numbers of non-BS-to-BS land cover transitions from coarser spatial and temporal resolutions to finer spatio-temporal resolutions using ancillary data (Nieves et al., 2020). This paper does not examine the built-settlement modelling framework in detail; see Nieves et al. (Nieves et al., 2020) for such details. However, we provide a description of the BSGM models to serve as background information. The intent is for readers to understand how these modelled BS extents, that are provided to the country-specific population models serving as the unit of analysis in this study, are constructed and influence end results.

**Fig. 2:**
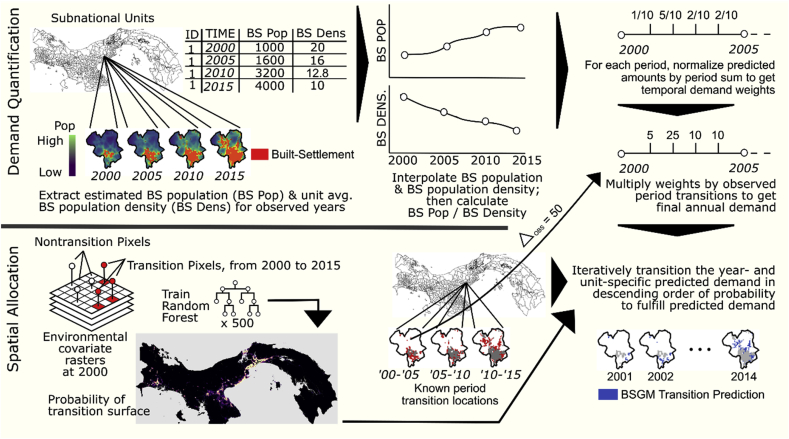
Generalized BSGMi process diagram from Nieves et al. (Nieves et al., 2020).

The BSGMi framework consists of two primary components: a Demand Quantification component and a Spatial Allocation component (Fig. 2) (Nieves et al., 2020).

Assume we are given a time period with at least two observations of BS extents, typically derived from remote sensing imagery, and corresponding estimated time- and unit-specific population found spatially coincident with the BS extents (Nieves et al., 2020). At regularly spaced intervals between the two or more observations, the BSGMi framework interpolates the BS population using unit-specific logistic growth curves to estimate unit-level BS population (Fig. 2) (Nieves et al., 2020). Similarly, the BSGMi uses natural cubic splines to interpolate unit-level changes in BS population density (Fig. 2) (Nieves et al., 2020). The BSGMi uses relative unit-level changes in interpolated BS population and BS population density to derive time- and unit-specific weights (representing unit-level non-BS-to-BS transition demand) (Nieves et al., 2020). These weights are utilized to temporally disaggregate the observed non-BS-to-BS transitions from the larger time period to the finer regularly spaced intervals, in this case years, between two or more observations (Fig. 2) (Nieves et al., 2020). This has the benefit of preserving agreement with the observed points (Mennis, 2003; Mennis & Hultgren, 2006; Nieves et al., 2020).

Once the number of transitions at the desired temporal level have been estimated, we move to the Spatial Allocation component of the BSGMi framework (Fig. 2) (Nieves et al., 2020). Here a Random Forest (RF) model (Breiman, 2001a; Liaw & Wiener, 2002), using predictive covariates listed in Table 1, predicted the pixel level probability of a non-BS-to-BS transition occurring between any two observed extent points (Nieves et al., 2020). This represents the period-level probability of transitioning and is further modified by using annual differences in lights-at-night (LAN) radiance values that are rescaled based upon the value distributions within their respective subnational units (Nieves et al., 2020). The values are rescaled, to values between 0 and 1, in such a way that pixels with greater unit-relative increases in LAN brightness are assumed to indicate a higher probability of transitioning and vice versa (Nieves et al., 2020). The RF pixel probabilities are multiplied by the corresponding LAN weights to produce year-specific probability surfaces that are then used, on a unit by unit basis, to iteratively disaggregate the year-specific predicted transitions, from the Demand Quantification component, across space (Fig. 2) (Nieves et al., 2020). However, given that the BSGMi is interpolative, transitions can only be allocated to pixels known to have transitioned in the period of interest (Nieves et al., 2020). Thus, the BSGMi produces a gridded time-series of BS spatial extents between every input, observed BS extents given (Fig. 2).

Previous validation of the BSGMi framework at 100m pixel resolution, given 4 observed years and predicting for twelve years, showed consistent performance across a variety of environments and contexts with the majority of interpolated years having a pixel level accuracy of greater than 80 percent (range 57–99 percent) (Nieves et al., 2020). However, the BSGMi framework utilized by the Global Project was an early version and differed from the version validated by Nieves et al. (Nieves et al., 2020) in two systemic ways: both the BS population and BS population densities were interpolated using unit-specific exponential growth/decay curves and the model was fit using only information from two time points at a time. This would likely result in an increased likelihood of overfitting for the BS population density across time, i.e. interpolated using information from two points rather than more than two, and a shifting of transitions to later in the time period due to the exponential curve shape. Nieves et al. (Nieves et al., 2020) found the model tended to predict transitions late so the latter, speculated, effect of having exponential assumption may mitigate this, but the magnitude and effect are unclear without further work. Further details of the potential implications of the early exponentially-based framework are given in a whitepaper produced by the WorldPop Group (Nieves, 2020).

Given that the BSGMi framework is top-down in nature, it is highly sensitive to the selected representation of BS selected as input (Nieves et al., 2020). Nieves et al. (Nieves et al., 2020) utilized the 300m ESA CCI “urban” land cover dataset, resampled to 100m, given its annual coverage allowed for holdout samples for validation. The population models from the Global Project utilized the BSGMi using a combination of GHSL and GUF data products, resampled to 100m, that were backfilled by the ESA CCI land cover data per Lloyd et al. (Lloyd et al., 2019). Despite these differences, the binary representation of the annual BS extents produced using the BSGMi were converted into a continuous representation of the Distance-To-nearest-Edge (DTE) of BS. This conversion to continuous distances and the fact the population models examined in this study are at the subnational unit-level, thus requiring us to take the unit-average DTE of BS, does effectively smooth any of the more frequent and smaller differences that would likely result, at various scales, due to the aforementioned differences between the validated BSGMi framework (Nieves et al., 2020) and the early, exponentially-based BSGMi framework (Nieves, 2020) BS extent predictions used in the production of the population models under analysis here.

#### 2.5.2 Top-down RF population disaggregation

**Fig. 3:**
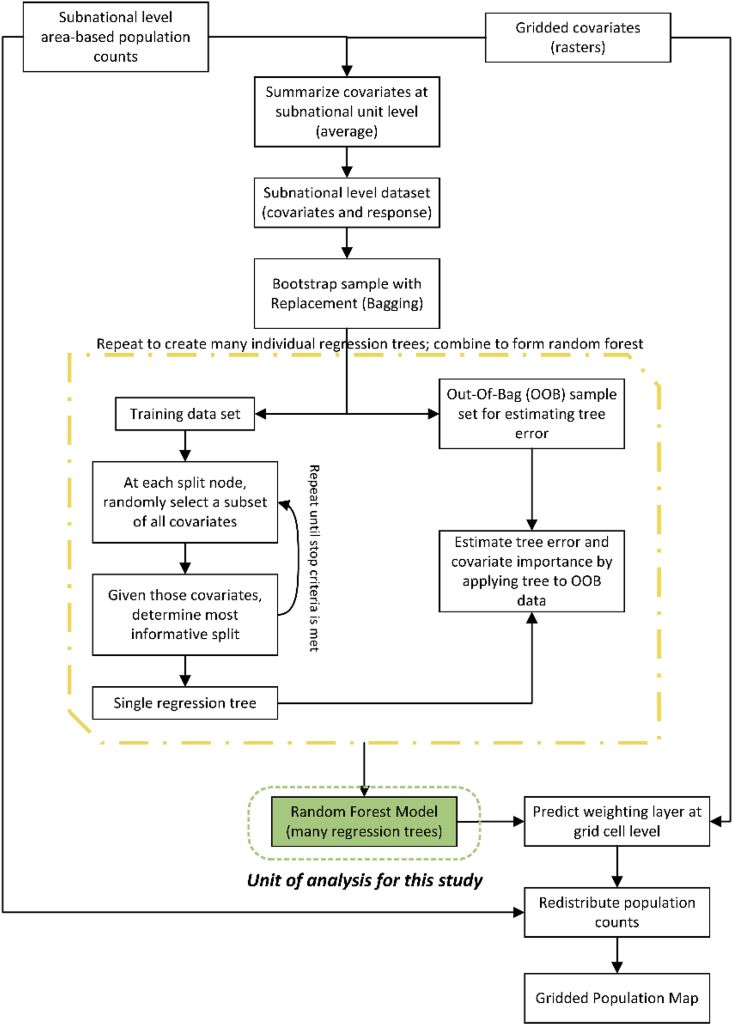
Generalized diagram of the RF-informed dasymetric disaggregation of population counts from subnational units to a given pixel level. Adapted from Nieves et al. (Nieves et al., 2017a).

The Global Project utilized a top-down RF informed dasymetric population disaggregation to distribute unit-level census-based population counts to pixel level (100m) population count estimates (Gaughan et al., 2016; Stevens et al., 2015; Gaughan et al., 2014; Sorichetta et al., 2015). RFs were chosen due to their automatability, scalability, ability to capture complex interactions and non-linear phenomena, and robustness to small samples and noise (Breiman, 2001a; Farror & Glauber, 1967; Rodriguez-Galiano, Ghimire, Rogan, Chica-Olmo, & Rigol-Sanchez, 2012). This modelling approach was applied on a country-by-country basis using a suite of globally harmonized and time-specific, or assumed temporally invariant, geospatial covariates which were aggregated by calculating the average of values within each subnational unit prior to being input to the RF (Fig. 3) (Gaughan et al., 2014; Gaughan et al., 2016; Sorichetta et al., 2015; Stevens et al., 2015).

While trained at the unit-level, using 500 trees, the RF is then used to predict population density at the pixel level (100m); we use these predictions as unit-relative weights to disaggregate the corresponding unit population count to pixel-level population counts while ensuring that the sum of pixel-level values sums up to the original unit-level count (Fig. 3) (Gaughan et al., 2014; Gaughan et al., 2016; Sorichetta et al., 2015; Stevens et al., 2015). Each year’s population disaggregation was done independently of the others.

RF models are a class of ensemble model where many “weak” classification and regression trees are combined through voting or averaging to produce more robust predictions (Breiman, 2001a). In this study, we utilize the *tunerf* function (Liaw & Wiener, 2002) to determine the optimal number of covariates to examine at each iterative split and carry out an iterative covariate selection process, per Stevens et al. (Stevens et al., 2015), to remove any covariates with an average Percent Increase in the Mean Squared Error (Per.Inc.MSE) less than or equal to zero (Stevens et al., 2015). The Per.Inc.MSE is an internal cross validation metric of covariate importance that is calculated by permutating the covariate information, preserving all other covariate information, and averaging the percent increase in the mean squared error across all trees in the RF when withheld “Out of Bag” (OOB) (Breiman, 1996; Breiman, 2001a) data is compared to the RF predictions. For further details on constructing RF models, bagging, and covariate selection and splitting in a random forest we refer readers to (Breiman, 1996; Breiman, 2001a; Liaw & Wiener, 2002; Strobl et al., 2007a; Strobl, Boulesteix, Kneib, Augustin, & Zeileis, 2008).

In general, the relative rankings of covariate importances within a RF are stable as long as several hundred trees have been grown (Breiman, 2001a; Dietterich, 2000; Strobl et al., 2008). However, Per.Inc.MSE is a relative, model specific, measure of importance that is highly conditional upon the other present covariates (Breiman, 2001a), presenting a challenge for using this metric when attempting to compare, even with a static set of covariates, the covariate importances across models (Nieves et al., 2017b). Additionally, while it is generally understood that the predictions of RFs are resilient to being provided correlated covariates (Breiman, 2001a), it does not preclude this correlation from affecting the relative covariate importances and covariate selection for splitting within a given model (Strobl et al., 2007a; Strobl et al., 2008). For instance, as is the case with the models examined here, if you have multiple representations of BS covariates in the model, with each covariate having partially overlapping fields of capture in the information space (i.e. they are correlated), and all are retained in the model, then the portions of the magnitude of the Per.Inc.MSE of will be “stolen” from the most important covariate (Breiman, 2001a). However, the relative ranking of the correlated covariates will be proportional to their frequency of utilization as splitting criteria across all trees, i.e. the most important covariate of the correlated covariates will still have the highest Per.Inc.MSE, it will just be of a smaller magnitude than without the inclusion of the correlated covariates in the RF.

### 2.6 Analyses

Our goal here was to capture the broad patterns of the relative rank of covariate’s importance across the globe based upon information contained within country-specific RF models used in disaggregating population. Given the potential difficulties of comparing covariate importance across independent RF models, we adopt the Weighted Importance Rank (WIR) from Nieves et al. (Nieves et al., 2017b) to facilitate our comparison of covariate importance across country- and time-specific RF population models. The WIR accounts for the potentially different number of covariates in each model, resulting from the covariate selection, by taking the ranking covariates within a given model by descending importance and dividing this rank by the total number of covariates in the model (Equation (1)) (Nieves et al., 2017b).

[1] $$WIR=\frac{within-model\;ranked\;importance}{total\;number\;of\;covariates\;in\;model}$$

This results in a value between 0 and 1, with the most important covariate having a value of 0 and the least important having a value of 1 (Nieves et al., 2017b). Hereafter, when referring to covariate importance, we are referring to the WIR as opposed to Per.Inc.MSE.

We collected all the RF model objects (*n ​=* 2236) produced in the modelling of population for the years 2000–2012, extracted the covariate importances (Per.Inc.MSE) into a data table, transformed the importances to WIR values, and assigned each country a label corresponding to their region (Fig. 1). Similar to Nieves et al. (Nieves et al., 2017b), we discovered the non-normal distributions of covariate importance data and, accordingly, adopted non-parametric statistical methods in conjunction with visual analyses. Using Kruskall-Wallis tests (Kruskal & Wallis, 1952; Rosner and Taylor, 2011), we tested for significant differences in the variable importance distributions of the BSGMi derived covariate: (i) between years 2001–2011 across all countries and, (ii), between countries grouped by regions (Fig. 1), across all years 2001–2011. Additionally, to determine if the annually modelled BSGMi-derived covariate was adding additional information to the models for years 2001–2011, we calculated the differences in WIR distributions: (i) between the annually modelled BSGMi-derived covariate and the historical BS extents at the year 2000 (GHSL-ESA 2000), (ii) between the annually modelled BSGMi-derived covariate and the annually available RS-derived “urban areas” extents (ESA Annual), and, (iii) between the historical BS extents (GHSL-ESA 2000) covariate and the annually available RS-derived (ESA Annual) covariate. Hereafter, we refer to the annually modelled BSGMi covariate, the historical BS extents covariate, and the annually available RS-derived covariates as the BSGMi, the GHSL-ESA 2000, and the ESA Annual extents. We then carried out one-sample Wilcoxon rank sum tests (Wilcoxon, 1945) to determine if there was a significant difference in the distributions of the WIR difference and a zero-median difference.

All Kruskall-Wallis and Wilcoxon rank sum tests were carried out with α ​= ​0.05 and, if significant results were found for the Kruskall-Wallis tests, these were followed up with *post hoc* Dunn tests with Holm correction for multiple outcomes (Dunn, 1964; Holm, 1979). Wilcoxon rank sum tests were adjusted for multiple outcomes as well using Holm’s correction. All models were carried out using the R statistical environment 3.4.2 (ore Team. R:anguag, 2017) and analyses were produced using the R statistical environment 3.6.0 (ore Team. R:anguag, 2019). All code, tabular data, and full test results are included in the supplementary materials.

## 3 Results

**Fig. 4:**
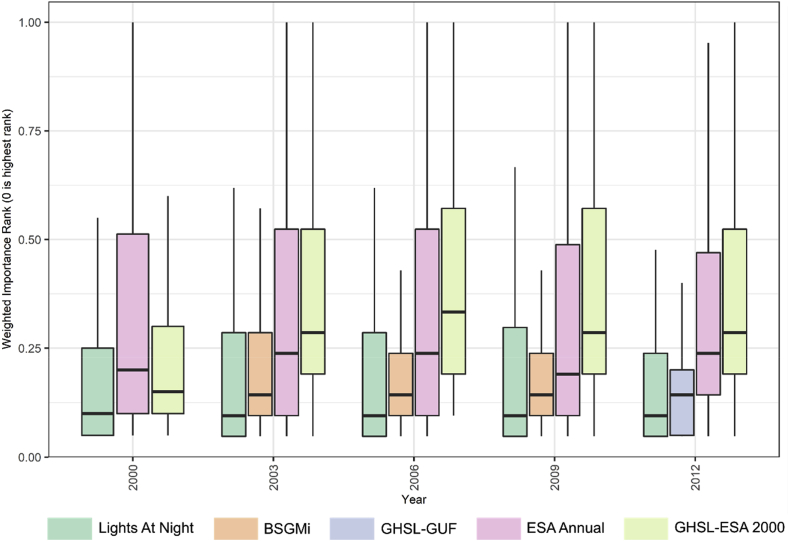
Boxplots of the weighted importance rank (WIR) of the four most important covariates in each year’s random forest model. WIR value distributions are shown for all countries by year with the median shown as a black line dividing the interquartile range (IQR, shown as the boxes) and 1.5 ∗ the IQR being represented by the “whiskers” of the plots.

Globally, across all years in the study period, we can see very consistent patterns of covariate importance. For clarity, we focus on five years (2000, 2003, 2006, 2009, 2012) and the four most important covariates (Lights-At-Night covariates, the BSGMi-derived covariate, the ESA Annual covariate, and the GHSL ESA 2000 covariate), hereafter. Based on the median WIR value, the lights-at-night (LAN) covariate is the most important covariate across all years (Fig. 4). For 2001 through 2011, the second, third, and fourth most important covariates are, respectively, the BSGMi-derived covariate (BSGMi), the ESA Annual covariate, and the GHSL-ESA 2000 covariate (Fig. 4). For the BSGMi covariate, we show that the variance decreases, and the median importance increases (smaller WIR value) with time, converging towards the 2012 GHSL GUF covariate’s distribution, which is what we would expect if the BSGMi model is interpolating accurately. Further, the distribution of the WIRs of the BSGMi-derived extents covariate appear to show consistency from one year to the next with an overall trend of decreasing WIR variance as the year becomes closer to 2012. At the global level, between years, there is no significant difference in the WIR distributions of the BSGM derived covariate (Χ^2^ ​= ​15.1, df ​= ​10, *p* ​= ​0.13; full results in supplementary materials).

**Fig. 5:**
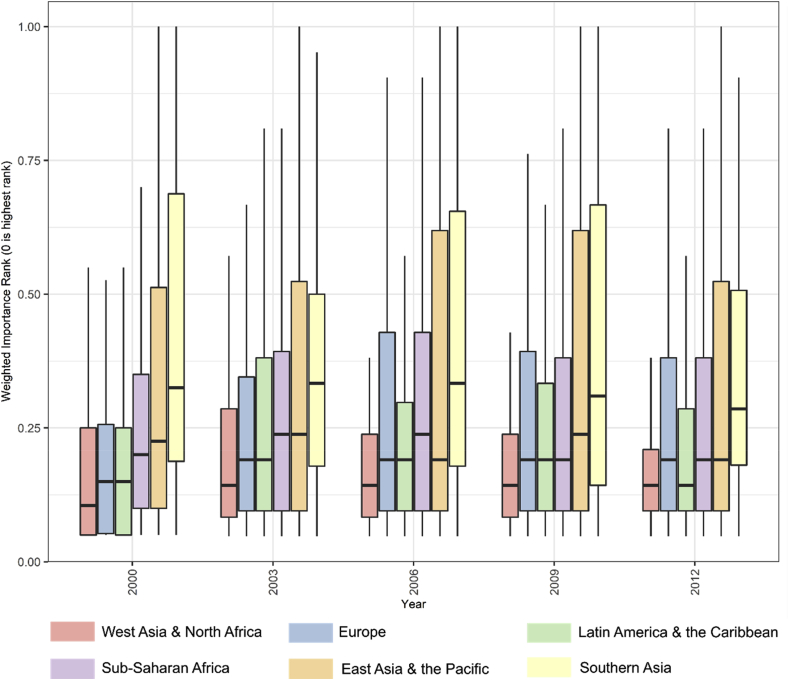
Boxplots of the weighted importance rank (WIR) of BS-related covariates in each countries’ random forest model, grouped by region and plotted by year with the median shown as a black line dividing the interquartile range (IQR, shown as the boxes) and 1.5 ∗ the IQR being represented by the “whiskers” of the plots. The BS-related covariate represented in 2000, 2003–2009, and 2012 are, respectively, the GHSL-ESA 2000 covariate, the BSGMi-derived covariate, and the GUF-GHSL covariate.

Looking only at the distributions of the BS-related covariates, we plotted the WIR boxplots by year and region in Fig. 5. Within a given region, it would appear there is generally consistent performance of the BSGMi-derived covariate with some regions exhibiting a slight temporal trend between 2000 and 2012, showing the large differences in GHSL dominated information (2000) and GUF dominated (2012) information provided to the RF (Fig. 5). A commonality, within most regions, would appear to be that the highest variance in WIR is seen near the midpoint of the interpolation period (2006) where we would expect performance of the BSGMi to be the worst or most variable (Fig. 5).

**Fig. 6:**
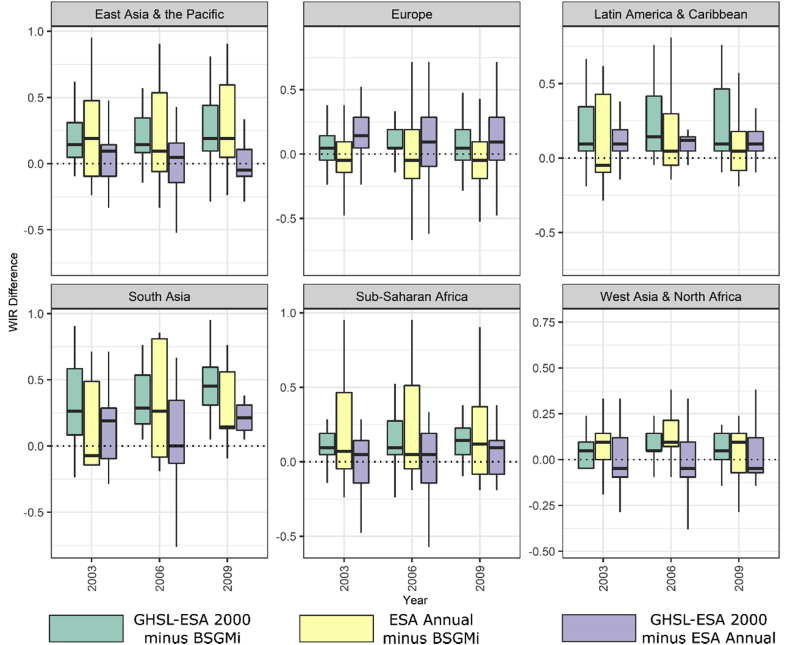
Box plot of WIR difference between the GHSL-ESA 2000 and the BSGMi-derived covariate, the ESA Annual and the BSGMi-derived covariate, and the GHSL-ESA 2000 and the ESA Annual covariates. For each comparison, positive WIR differences indicate that the former of the pair was less important than the latter and negative values indicate the opposite. Results for all years are included in the supplementary materials.

We plotted the WIR difference between all pairwise combinations of the three covariates of interest and tested their distributions, across all years for each region, to determine if they were significantly different from a distribution with a median WIR difference of 0, i.e. the covariates contribute the same amount of importance (Fig. 6, Table 3). When testing for significance, data were aggregated across years 2001–2011 and grouped by region. We show that across all regions the annual BSGMi covariate was contributing significantly more importance (*p* ​< ​0.00 for all regions) to the RF model than the “historical” GHSL-ESA 2000 covariate. The largest difference for this is seen in the “South Asia” and “East Asia & the Pacific” regions. When compared to the ESA Annual covariate, the BSGMi covariate is contributing significantly more importance to the RF model in all regions (*p* ​< ​0.00) except “Europe” (*p* ​= ​0.99). Examining the differences between the GHSL-ESA 2000 and the ESA Annual WIR values, we see that the ESA Annual data is contributing significantly more importance in all regions (*p ​< ​0.00*) except the “East Asia & the Pacific” (*p* ​= ​0.14) and the “West Asia & North Africa” regions (*p* ​= ​0.77).

**Table 3:** Adjusted p-values of Wilcoxon one sample test with Holm correction for examining significant differences in covariate importance as measured by the Weighted Importance Rank (WIR). Data was aggregated across years 2001–2011 and grouped by region. Null hypothesis being that the median WIR difference of a given comparison was equal to zero. Significant differences are shaded for emphasis. Full results are provided in the supplementary materials.

WIR Differences	East Asia & the Pacific	Europe	Latin America & the Caribbean	Southern Asia	Sub-Saharan Africa	West Asia & North Africa
GHSL ESA 2000 minus BSGMi	<0.00	<0.00	<0.00	<0.00	<0.00	<0.00
ESA Annual minus BSGMi	<0.00	0.99	<0.00	<0.00	<0.00	<0.00
GHSL ESA 2000 minus ESA Annual	0.14	<0.00	<0.00	<0.00	<0.00	0.77

## 4 Discussion

We have shown that interpolated year-specific BS-extent data, using the BSGMi framework, is a consistently important predictor of population density globally and across time. Specifically, the BSGMi-derived covariate was consistently second most important, behind year-specific lights at night data. Even though both the lights at night data and the BSGMi data are given to the model as continuous covariates. Essentially, the BS-derived covariates only indicate presence and absence of BS while lights at night can capture presence, absence, and intensity of BS presence (Small, Elvidge, Balk, & Montgomery, 2011). This is not to say that LAN are inherently superior to BS datasets as LAN can capture lights that have little to do with the definition of BS or indicating where people reside (e.g. parking lots, green houses, lit highways). The annually available RS-based BS representation (ESA Annual) and the “historical” single year RS-based BS covariate (GHSL-ESA 2000) are still highly important within the models (Fig. 4, Fig. 5) even with the presence of the LAN data and it is important to remember that the covariate importances are conditional upon the given set of covariates (Breiman, 2001a). Additionally, the ESA Annual and GHSL-ESA 2000 covariates can give relative indications of how the chosen BS representation and the BSGMi perform within regions. However, for any given region, these differences in importance were stable across time (Fig. 6). Overall, BSGMi interpolated extents increase the information in these population models and, combined with the other RS-derived covariates, likely better capture the BS-information space as related to population density than any one covariate does alone.

Regardless of the magnitude of the importance or relative importance, a key point is that the BSGMi-derived covariate was always retained in models that it was introduced to and consistently contributed significantly more importance to the models than the other BS representations, across most regions. The fact that all of the representations of BS were consistently the 2nd through 4th most important covariates across all years supports previous importance findings (Nieves et al., 2017b) and reemphasizes that utilizing multiple representations of BS results in more accurate disaggregative population modelling (Reed et al., 2018).

We would expect a year-specific BS covariate to contribute significantly more information than a “historical” BS covariate, which was largely supported by the findings in Fig. 6 and Table 3tbl3. However, historical extents can be critical when modelling populations across time (Gaughan et al., 2016). The exceptions of year-specific BS dominance in “East Asia & the Pacific” and “West Asia & North Africa” could be explained by several factors: (i) large and or few subnational units, (ii) lack of suitable, e.g. cloud free imagery for these optically based datasets, and/or, (iii) greater difficulty in urban feature extraction within arid regions (i.e. similar radiometric signature between buildings and bare soil) contributing to greater noise in the population density-BS relationship fit by the RF. This could potentially explain the relatively poorer importance contribution of the BSGMi covariate in the “East Asia and the Pacific” and the “South Asia” regions (Fig. 5). Additionally, it is important to note that this study uses the original GHSL as a part of its input BS representation and, therefore, it is currently unclear if the newer versions (Corbane et al., 2017a), which leverage the increased resolution and different radiometric capture of the Sentinel platforms, would change these findings (Fig. 6 and Table 3). The other notable result of Fig. 6 and Table 3 is the lack of significant difference between the ESA Annual covariate and the BSGMi covariate. This could be potentially explained by: (i) the ESA data does rather well within Europe’s dense and well-defined BS extents, and, (ii) those BS extents do not change as much as other regions, i.e. the non-BS to-BS transition prevalence is low so the BSGMi model does relatively worse than in a high transition area (Nieves et al., 2020). Regardless, it is important to note that the results of Fig. 6 and Table 3 are relative and that all the covariate representations of BS were found to be important to the RF model of population density.

From previous work (Nieves et al., 2020), there is little doubt that the BSGMi is picking up true BS extents that, in turn, drive this increased importance. However, the regional differences can more generally be attributed to the chosen RS-derived BS extents input into the BSGMi framework, the quality of the input population data, and the size and configuration of the subnational units used in both the BSGMi and the population modelling method used here (Nieves et al., 2017b; Nieves et al., 2020; Openshaw, 1984; Stevens et al., 2015). To investigate if different underlying structures of causal relationships between population and BS exist, and to then quantify them, a different research framework and modelling approach, i.e. an explanatory modelling framework as opposed to a predictive one (Breiman, 2001b; Shmueli, 2010), would be necessary.

Nieves et al. (Nieves et al., 2020) suggested that end users of the BSGMi modelling framework check the model outputs for end use suitability and accuracy. The regional differences in the WIR of the BSGMi-derived covariates (Fig. 5) reinforce that it is important that users of any modelled BS extents examine them for their use-specific and study area-specific suitability as no model framework is likely to excel in all scenarios. These observed WIR differences can be due to pre-existing differences in the suitability of the input BS representation or due to model-induced uncertainty and error, but in an applied context, the origin is of secondary importance to knowing of its existence.

These findings are for these specific representations of BS and the importances are contingent upon the set of covariates provided (Breiman, 2001a). We would hypothesize that if we were to include the BSGMi-derived covariate as the only representation of BS in the RF models, acknowledging that within a RF correlated variables “take” importance away from each other, there is a possibility that it could surpass the LAN covariate for most important, but this awaits further study. Further, while here we explored the importance of the BSGMi-derived and other BS-based covariates at the subnational unit level, how this subnational importance translates into the accuracy of the disaggregated, i.e. pixel level, population maps produced using weights derived from the RF that contains the modelled BS-extents is something that is still an open question. This is because as pixel level (~100m) population data is often not available for validation purposes (Sinha et al., 2019). We would like to think that having more important covariates at the subnational level would result in more accurate pixel-level disaggregations, but the issues of scale and other inputs into the model make any speculation tenuous, at best.

As previously noted, while RF predictions are resilient to correlated variables that does not imply that RFs are impervious to issues of correlated variables or spatial autocorrelation in the data. One investigation on RF-informed population disaggregations found that spatial autocorrelation of the residuals at the subnational unit scale, i.e. the spatial scale of RF training, is more so an effect of the ensemble nature of the RF, which cannot predict outside the observed range of the response variable (Sinha et al., 2019). This, on average, causes urbanized areas to be underpredicted and rural areas to be overpredicted (Sinha et al., 2019; Stevens et al., 2015). This showed that when autocorrelation was relatively low, the out-of-bag error of RFs was similar to that calculated using a holdout sample and, when autocorrelation was relatively high, the holdout samples showed lower error than the out-of-bag estimates (Sinha et al., 2019). This does have potential implications for the covariate importances, e.g. covariates importances estimated via bagging could be overestimated, however a separate research question and framework would be needed to interrogate this. There is also evidence that, within a RF, correlated variables are more likely to be selected as important, which has led to the creation of different RF versions that attempt to account for this (Strobl et al., 2007a; Strobl et al., 2007b; Strobl et al., 2008). However, no direct comparison of the outcomes of, say, a RF constructed using conditional variable importance against the standard RF implementation used here. These are all worth future exploration as they could give indication into potential subnational biases or variation in the redistributions of population counts.

While suited for the objectives here, the WIR is a rather limited metric in that it only captures the coarse patterns of variable importance at the level of representation in the model, i.e. national level. This obfuscates any potential subnational variation in covariate importance and or contribution to predicted populations. Alternative metrics such as Accumulated Local Effect plots (Apley & Zhu, 1612), Shapley values (Cohen, Ruppin, & Dror, 2005; Shapley, 1957), and others can provide more insight into the contribution of different covariates and individual observations into the model’s predictions. However, these metrics can be expensive to compute, require special consideration when applied to correlated data, and require access to all of the model’s training data. Regardless, metrics such as these should be considered and calculated at the time of model training in order to facilitate better understanding of model and data behavior, as well as with the foresight of better facilitating secondary analyses.

The Nieves et al. (Nieves et al., 2020) validation of the BSGMi framework was with an originally coarser representation of BS (300m ESA CCI landcover) and the authors queried whether the assumed relationships of the framework would hold with originally finer scale input BS extents given their findings and previous findings under a different framework (Tayyebi et al., 2013). While this study does not perform a pixel-based validation of the BSGMi, here we have shown that using originally finer scale input BS extents can produce derived data products that were found to be informative for applications, causing us to speculate that the framework assumptions do hold. However, whether that indicates the pixel-level BSGMi outputs can be utilized without aggregation, as we have done here for our end use, remains unclear.

Within the population models analyzed here, the single year BS extents representing historical BS extents was limited to the year 2000. Therefore, our findings related to importance as compared to the historical extents would likely change, at a minimum, in magnitude were the historical extents year to be different, dynamic, or to include multiple historic BS extents. While Gaughan et al. (Gaughan et al., 2016) found that including previous BS extents were important in creating temporally comparable population surfaces when performing top-down modelling, there is no current information regarding at what temporal lag the information contributed is maximized and how many previous representations should be included.

## 5 Conclusions

Here we tested the utility of the modelled BS extents in a population-modelling scenario across 172 countries and 13 years. Globally, we found that modelled BS extents are consistently the second most important predictor of population density, even when the previous RS-derived BS extents and time-specific BS-extents were included in the model. However, regional variation exists in the importance of the modelled BS extents, but its cause is multifactorial and still unclear. Additionally, there were many cases where the time-specific RS-derived covariate, originally having a coarser spatial resolution, was more important than the high-resolution modelled BS extents and/or the high-resolution previously observed RS-derived extents. Combined with the fact that all covariates were retained in the final models, this would suggest that while modelled BS extents are informative, they are best used in conjunction with other representations of BS when modelling population.

These findings are specific to the spatial scale and zonal configuration of the subnational units used. Future work examining the impact of the scale of the subnational units on both the BS modelling and RF-informed dasymetric modelling should be conducted, although some previous work would indicate that smaller units leads to more accurate models (Gaughan et al., 2014). While this study has shown that the BS modelled extents are important at the subnational unit level, future work should examine how the BS modelled extents affect the pixel level predictions and smaller area population predictions in this top-down modelling framework. Additionally, research into the number of previous extents to include in the population modelling as well as the effect of its temporal lag on population predictions should be investigated.

## Funding

JJN is funded through the Economic and Social Research Council’s Doctoral Training Program, specifically under the South Coast branch (ESRC SC DTP). AS is supported by funding from the 10.13039/100000865Bill & Melinda Gates Foundation (OPP1134076).

## CRediT authorship contribution statement

**Jeremiah J. Nieves:** Conceptualization, Data curation, Formal analysis, Funding acquisition, Investigation, Methodology, Project administration, Software, Resources, Validation, Visualization, Writing - original draft, Writing - review & editing, Data curation, Investigation, Resources, Software, Writing - review & editing. **Maksym Bondarenko:** Data curation, Investigation, Writing - review & editing. **David Kerr:** Data curation, Investigation, Writing - review & editing. **Nikolas Ves:** Data curation, Investigation, Writing - review & editing. **Greg Yetman:** Data curation, Investigation, Writing - review & editing. **Parmanand Sinha:** Conceptualization, Resources, Supervision, Writing - review & editing. **Donna J. Clarke:** Conceptualization, Funding acquisition, Resources, Supervision, Writing - review & editing. **Alessandro Sorichetta:** Conceptualization, Writing - review & editing. **Forrest R. Stevens:** Conceptualization, Writing - review & editing. **Andrea E. Gaughan:** Conceptualization, Supervision, Resources, Funding acquisition, Writing - review & editing.

## Declaration of competing interest

The authors declare no conflict of interest. The funders had no role in the design of the study; in the collection, analyses, or interpretation of the data; in the writing of the manuscript, or in the decision to publish the results.

**Table 1:** List of countries modelled. Countries are given by their ISO standard 3-letter code.

Region	Countries (ISO 3 Code)
East Asia & the Pacific	ASM AUS BRN CHN FJI FSM GUM HKG IDN JPN KHM KIR KOR LAO MMR MNG MNP MYS NCL NZL PHL PNG PRK PYF SGP SLB THA TLS TUV TWN VNM VUT WSM
Europe	ALB ARM AUT AZE BEL BGR BIH BLR CHE CYP CZE DEU DNK ESP EST FIN FRA FRO GBR GEO GRC HRV HUN IRL ISL ITA KOS LTU LUX LVA MDA MKD MLT NLD NOR POL PRT ROU RUS SRB SVK SVN SWE TUR UKR
Latin America & the Caribbean	ABW ARG BOL BRA CHL COL CRI CUB CUW DOM ECU GTM GUY HND HTI MEX MTQ NIC PAN PER PRI PRY SLV SUR URY VEN
South Asia	AFG BGD BTN IND LKA MDV NPL PAK
Sub-Saharan Africa	AGO BDI BEN BFA CAF CIV CMR COD ETH GAB GHA GIN GMB GNB KEN LBR LSO MDG MLI MOZ MRT MUS MWI NAM NER NGA RWA SEN SLE SOM SWZ SYC TCD TGO TZA UGA ZAF ZMB ZWE
West Asia & Northern Africa	DZA EGY IRN IRQ ISR JOR KAZ KGZ LBN MAR OMN QAT SAU SDN SSD SYR TJK TUN YEM

**Table 2:** List of countries excluded from analysis and corresponding reason for exclusion

Countries Excluded	Reason for Exclusion
Antarctica	Not modelled at all
United States of America	Resource limits
Anguilla; Aland Islands; Andorra; United Arab Emirates; Antigua and Barbuda; Bonaire, Sint Eustatius, and Saba; Bahrain; Bahamas; Saint Barthelemy; Belize; Bermuda; Barbados; Botswana; Republic of Congo; Cook Islands; Comoros; Cape Verde; Cayman Islands; Djibouti; Dominica; Eritrea; Western Sahara; Falkland Islands; Guernsey; Gibraltar; Guadeloupe; Equatorial Guinea; Grenada; French Guiana; Isle of Man; Jamaica; Saint Kitts and Nevis; Kuwait; Libya; Saint Lucia; Lichtenstein; Macao; Saint Martin (French portion); Monaco; Marshall Islands; Montenegro; Montserrat; Mayotte; New Caledonia; Norfolk Island; Niue; Nauru; Pitcairn Islands; Palau; Palestine; Reunion; Saint Helena; Svalbard and Jan Mayen Islands; San Marino; Saint Pierre and Miquelon; Sao Tome and Principe; Sint Maarten (Dutch portion); Seychelles; Turks and Caicos Islands; Tokelau; Turkmenistan; Tonga; Trinidad and Tobago; Vatican City; Saint Vincent and the Grenadines; British Virgin Islands;Virgin Islands (U.S.); Wallis and Futuna	Regional parameterization of BSGM and or population model
